# Schooling, Local Knowledge and Working Memory: A Study among Three Contemporary Hunter-Gatherer Societies

**DOI:** 10.1371/journal.pone.0145265

**Published:** 2016-01-06

**Authors:** Victoria Reyes-García, Aili Pyhälä, Isabel Díaz-Reviriego, Romain Duda, Álvaro Fernández-Llamazares, Sandrine Gallois, Maximilien Guèze, Lucentezza Napitupulu

**Affiliations:** 1 Institució Catalana de Recerca i Estudis Avançats (ICREA), Barcelona, Spain; 2 Institut de Ciència i Tecnologia Ambientals, Universitat Autònoma de Barcelona, Cerdanyola del Vallès, Barcelona, Spain; 3 Department of Biosciences, University of Helsinki, Helsinki, Finland; 4 Museum National d'Histoire Naturelle, Site du Musée de l’Homme, Paris, France; The University of Chicago, UNITED STATES

## Abstract

Researchers have analysed whether school and local knowledge complement or substitute each other, but have paid less attention to whether those two learning models use different cognitive strategies. In this study, we use data collected among three contemporary hunter-gatherer societies with relatively low levels of exposure to schooling yet with high levels of local ecological knowledge to test the association between *i)* schooling and *ii)* local ecological knowledge and verbal working memory. Participants include 94 people (24 Baka, 25 Punan, and 45 Tsimane’) from whom we collected information on 1) schooling and school related skills (i.e., literacy and numeracy), 2) local knowledge and skills related to hunting and medicinal plants, and 3) working memory. To assess working memory, we applied a multi-trial free recall using words relevant to each cultural setting. People with and without schooling have similar levels of accurate and inaccurate recall, although they differ in their strategies to organize recall: people with schooling have higher results for serial clustering, suggesting better learning with repetition, whereas people without schooling have higher results for semantic clustering, suggesting they organize recall around semantically meaningful categories. Individual levels of local ecological knowledge are not related to accurate recall or organization recall, arguably due to overall high levels of local ecological knowledge. While schooling seems to favour some organization strategies this might come at the expense of some other organization strategies.

## Introduction

Research suggests that schooling can affect strategies used to recall information, by changing the brain organization of cognition in a myriad of ways, but researchers debate whether such cognitive effects systematically relate to everyday problem-solving [1]. Thus, although several studies show that schooling and the acquisition of school-related skills can have lasting positive effects on some cognitive and non-cognitive skills [2–4], it is also possible that school-based learning has no effect, or even negative effects, on more procedural, pragmatic and sensory oriented types of learning also needed for everyday life. This could be particularly the case in situations where everyday activities do not necessarily depend on the type of cognitive skills learned at schools, as is often the case among small-scale, rural populations. Ardila and colleagues [4] point that contemporary illiteracy is not the same as preliteracy, as literacy might have replaced preliterate cognitive skills. Preliterate societies often have rich cultural traditions, largely passed by oral means which might require specific cognitive skills, likely different than those used in school.

Indeed, for most of human history, the acquisition of knowledge and skills needed for subsistence did not occur in school, nor did it depend on the skills and behaviours learned in the classroom. Rather, the learning of subsistence skills occurred through participation in everyday activities in which local knowledge of plants, animals, and the environment was socially transmitted [5]. Research suggests that, even today, in indigenous societies local ecological knowledge produces positive returns to the individual [6, 7] in the same way that schooling and the skills and behaviours learned in school do [8, 9]. For example, researchers have found that local ecological knowledge helps indigenous societies deal with pest infestations [10], cope with weather variability and adapt to climatic change [11], select cultivars [12], manage natural resources [13], and enhance health and nutritional status [6].

Cognitive psychologists and anthropologists have analysed the associations between school and local ecological knowledge, suggesting that among indigenous groups, schooling and academic skills learned in school undermine local ecological knowledge, arguably because time and resources spent in school detract from time and resources spent learning other forms of knowledge [14–16]. But, to date, we lack research focussing on whether those two models of knowledge acquisition have a differentiated impact on cognition.

We argue that this could be the case as both systems use different learning strategies and display critical differences in the social organization of learning [17]. Schools represent a special type of setting centered on student´s learning, reasoning, comprehension, memorizing, problem solving, and achieving. Therefore, attending school contributes to the development of specific attitudes towards knowing, understanding, and thinking. Indeed, long ago, Bartlett [18] proposed that literate people might use more active information integration procedures (or “meta-memory”) than illiterate people, who in turn might more frequently use rote learning (or memorizing by repetition). Since then, several studies have shown that school-attendance, as well as the acquisition of skills typically learned at school (e.g., literacy and numeracy) affect cognition and cognitive abilities [19]. Even one or two years of school can affect performance in certain neuropsychological tests, differences being more significant during the first three years of education, followed by a negatively accelerated curve tending to plateau [20]. Additionally, some skills learned in school, such as literacy, are effective tools for acquiring information from any sources (books, magazines or journals), which might be contrasted with information obtained from oral sources or own experience, potentially altering the way the individual conceptualizes and interprets the world [4].

In contrast, children usually acquire local knowledge from early childhood [21], from many social settings and “teachers” [22], and largely through observation, imitation and emulation during the performance of daily life activities [5, 23, 24]. Furthermore, rather than relying in specialized child-focused activities to teach children, the acquisition of local knowledge is largely self-motivated [17, 25].

While the two ways of learning are not necessarily opposite, the prevalence of one may have impacts on people’s cognition.People without schooling might favour different types of learning or different learning strategies helping them learn and remember in ways that are relevant for their everyday lives. Moreover, some research suggests that such alternative strategies are not well captured in standard tests. For example, in a classic study on the effects of schooling on memory, Cole and Scribner [26] found differences in memory performance between Liberian children who attended school and those who did not when using a standard memory test. However, when non-schooled children were tested using a story, their recall accuracy improved, as they were able to recall the objects by associating them with the roles they played in the story. Similar results have been found on memory tests in research among Mayan children of rural Guatemala [27].

Here, we test the differentiated association of *i)* schooling and *ii)* local ecological knowledge on verbal working memory using data collected among three contemporary hunter-gatherer populations with relatively low levels of exposure to formal schooling, yet with high levels of local ecological knowledge. We focus on verbal working memory (or the ability to remember information in the form of words) for two main reasons. First, the verbal working memory system allows people to hold a small set of representations active for a short period of time [28, 29]. This ability is critical for many higher-level cognitive abilities, including learning and understanding language, manipulating numerical representations, inferring the causal structure of events, and understanding social interactions [29]. So, working memory is a critical cognitive skill for the acquisition of any type of knowledge. Second, many of the participants in our study were not only illiterate but–according to our field experience- some of them were also unfamiliar with printed materials in general, and we were afraid they will have problems identifying objects in a photograph. So we considered that verbal tasks were better accepted and potentially less biased than tasks involving printed material.

Researchers differentiate between school attendance and the acquisition of skills typically learned in school as the two phenomena do not necessarily correlate [4, 30]. Similarly, researchers studying local knowledge systems have differentiated between theoretical (or declarative) knowledge and practical skills [31, 32]. We test whether standard measures of performance on a verbal working memory task relate to a) schooling, b) skills typically learned in school (literacy, numeracy), c) local ecological knowledge related to hunting and medicinal plants, and d) skills related to hunting and medicinal plants.

## The Setting

We collected data in three indigenous, small-scale, subsistence-based societies with little involvement in market economies, school-based education, or modern healthcare systems: the Baka (Congo Basin), the Punan (Borneo), and the Tsimane’ (Amazonia). The three societies resemble one another in that they depend on the consumption of local and self-harvested natural resources for their subsistence, generally based on a combination of foraging and farming and in that they have only recently started to integrate more regularly with the broader society, with only relatively recent adoption of western-style classroom schooling. Furthermore, as for many other indigenous populations [33, 34], the quality and rigour of schooling in the studied societies is relatively limited. Below we provide some glimpses of each of the three societies, with special emphasis on access to schooling.

The Baka are one of the several hunter-gatherer groups living in the tropical rain forests of the Congo Basin. Their population was estimated twenty years ago to be about 26000 individuals in Cameroon, spread in more than 400 villages [35]. As other hunter-gatherers in the region, the Baka traditionally depended on wild animals and plants for their livelihoods, although they also maintained economic and social relations with sedentary farming villagers, with whom they exchanged forest products for agricultural ones and to whom they occasionally provided agricultural manual labour [36]. Baka subsistence activities changed at the turn of the 1960s, when they began to settle along new logging roads and to cultivate their own plots [35]. Nowadays, the Baka combine hunting-gathering with cultivation of cassava and plantain, their major staple crops, alongside their menial work on the farms of their Bantu neighbours. While no longer nomadic, the Baka still spend long periods of time in forest camps, where their subsistence depends on hunting and gathering.

Schooling is relatively recent for the Baka. Some 25 years-ago, Bahuchet [37] reported that less than 5% of Baka children were registered at school. Since then, and concomitant with sedentarization efforts, there have been several efforts to promote schooling among the Baka, yet numbers remain low, and schooling generally only covers primary education. Missionaries and local NGOs have trained teachers and established private schools reserved for Baka people in which teaching is carried out at least partly in the Baka language. In communities without access to Baka-speaking schools, Baka children can attend public schools typically established in their Bantu-speaking neighbouring villages. In such cases, however, Baka children face important barriers to schooling, including registration fees, discrimination on the base of their ethnic origin, and the use of French as language of instruction.

Our second study society, the Punan, is a group of ~10000 people living in Indonesian Borneo [38]. Their traditional economy was based on hunting bearded pigs and preparing starch from hill sago, a wild clump-forming palm. They have a long history of barter with the locally settled farmers [38]. Efforts to sedentarize the Punan started in the mid-1950s [39]. Nowadays, the Punan are no longer a nomadic group, but they still engage in long travels and seasonal stays in the forest for hunting wild boars and mostly for gathering forest products which can be commercialized, such as eaglewood [38, 39]. The Punan are undergoing many social and economic changes, including increasing engagement in wage-labour, adoption of swidden rice cultivation, and dependence on government subsidies. Collecting and trading forest products such as eaglewood, rattan, and live animals are important income-generating activities.

The first effort to provide schooling to the Punan dates four decades back. In 1973, the then dictatorial national government initiated a massive national schooling project aiming at raising national loyalty for which they concentrated in spreading the use of Bahasa [40]. Elementary schools, where teaching was done in Bahasa (Indonesian national language), were opened in all sub-districts, although secondary education was generally still out of reach for rural populations [41]. After the downfall of the dictatorship, the 1998 Decentralization Law put regional development (especially in remote areas) as a national priority. Limberg and colleagues [42] reported the building of the first elementary school in the most remote Punan villages in 2002. Today, Punan children have relatively easy access to formal schooling at the elementary level, although the overall attendance rate is low and teachers’ absenteeism is high. Secondary education is still largely unavailable; the first remote high school was opened in 2013, before which secondary education was available only in regional towns [39]. Despite these limitations, parents will send their children to the towns. Schooling is increasingly becoming a priority for Punan parents, even to those living in remote villages, as they perceive that education can provide children with job opportunities [38].

Our third study society are the Tsimane’, a small-scale indigenous society of foragers and farmers in the Bolivian Amazon. The Tsimane’ number ~ 12,000 people living in ~100 villages of ~20 households, concentrated along rivers and logging roads. Up until the late 1930s, the Tsimane’ maintained a traditional and self-sufficient lifestyle, but their interactions with the Bolivian society have steadily increased since the 1940s [43]. Previously semi-nomadic, they are now mostly settled in permanent villages with school facilities. The Tsimane’ rely on slash-and-burn farming supplemented by hunting, fishing, gathering, and wage labour in logging camps, cattle ranches, and in the homesteads of colonist farmers. Their main cash crops are rice and maize [44], although the barter of thatch palm also provides an important source of income for many households.

The Tsimane’ have been exposed to schooling since the 1950s, when the Bolivian government gave Protestant missionaries the responsibility for schooling remote lowland native Amazonian populations [45]. The missionaries trained young Tsimane’ men who later became teachers. These teachers provided schooling in 80% of Tsimane’ villages (the rest having no schooling) until 2006, providing a partially contextualized primary education curriculum (i.e. teaching in Tsimane’ language and using examples from the local environment) [15]. However, since 2006, the new Bolivian Education Law has homogenized the content of school curricula, imposing Spanish as the vehicular language [46]. The effort to improve overall education to lowland populations is also leading to the replacement of the missionaries-trained Tsimane’ teachers by national university-trained teachers, who typically come from different cultural contexts (i.e., Bolivian highlands) and do not speak the Tsimane’ language, and consequently have difficulties to adapt to the lowland ecological and social context [47].

In sum, in the three studied societies, access to the school system is relatively recent and, because school attendance was -in practice- voluntary, many adults of the current generation have never attended school. Furthermore, in none of the studied societies are the skills learned in school necessary for subsistence, which in turn depends on the acquisition of local environmental knowledge transmitted orally and through the context and acquired by observation and practice (see [48] for a description of Baka local ecological knowledge, [38] for the Punan, and [49] for the Tsimane’). Such settings thus offer the possibility to test whether the two differentiated ways of learning, schooling and local knowledge, differently relate to cognition.

## Methods

We set up data collection within the framework of a larger research project [50] for which six researchers conducted 18 months of fieldwork each. To work in the Tsimane’ territory, we obtained written permission of the Great Tsimane’ Council. To work among the Punan, we obtained permission from RISTEK (Ministry of State for Research and Technology, Indonesia, SIP NO: 038/SIP/FRP/SM/II/2012). No specific permissions were required to work in the area where the Baka live. During the first months of fieldwork, they learned the local languages, adapted to the local mores, build up trust with participants, collected background information, and developed the methodologies to be used later. Data on schooling and school-related abilities were collected at the beginning of fieldwork and updated later. Data collection on local knowledge and related skills took place over the course of 12 consecutive months, during which researchers visited each informant several times. Memory data were collected in the last three months of field research, once informants were well familiar with the researchers. All interviews were done in the local language, with the support of trained field assistants.

### Participants

Participants were recruited among adults in the framework study [50] and participation was strictly voluntary. As the Tsimane’ have a political organization that represents them, we obtained written agreement from this organization (The Great Tsimane’ Council). In the three settings we obtained written consent from the head of the village and oral Free Prior and Informed Consent from each individual participating in the study. We opted for oral consent because part of the target populations had little schooling and very little exposure to Western customs that involve mastery of concepts such as agreements, legal contracts, or ‘informed consent’. Consequently many did not understand well the implications of signing a form. In contrast, they are comfortable providing informed consent verbally. Participants include 94 adults, considered here as people over 16 years of age (44 women and 50 men) living in the three mentioned forager societies (24 Baka, 25 Punan, and 45 Tsimane’). For participants under 18 years of age, we obtained oral informed consent of one of the parents. The ethics committee of the Autonomous University of Barcelona (CEEAH-04102010) approved the protocol for this research, including the collection of oral consent. Oral consent was documented during the first interview (a census). Due to high mobility, we could not collect data for all informants and all the tasks, so there are slight sample size variations (± 8 subjects) in some tests.

### School and school-related skills

We collected school-related information of each participant. We asked informants about the maximum school grade they had attained. Since, overall, levels of school achievement are low, and as in some of the communities some individuals acquire or improve their school-related skills outside the school (e.g. basic numeracy can be improved when negotiating prices with vendors or traders), we also conducted direct measures on literacy and numeracy. We assessed each informant’s literacy by requesting them to read a simple sentence. We had sentences in native languages and in the national languages and used the one in which the informant performed best to assign a score. We coded answers for literacy tests as 0 = unable, 1 = with difficulty, 2 = well, but because low variation in data, we later grouped people who read (either with difficulty or well) in a single group as opposed to people who could not read at all. To assess numeracy we asked informants to perform four elementary calculations (adding, subtraction, multiplication, and division). We assigned a 1 to each correct answer and stopped the test if the person made an error. Thus, numeracy could range from zero to four. We had three equally difficult versions of the literacy and numeracy tests and chose one at random for each person.

### Local ecological knowledge and skills

Due to the variations in local environments and knowledge between our three case study sites, we had to develop site-specific knowledge tests for each. To allow for the comparability of data across sites, we followed the same protocol, including the way in which questions were generated and the general structure of the tests (see [51] for detailed description). We assessed the local knowledge of a person through four tests. The first test measured the person’s knowledge of medicinal plants and consisted of an identification task, in which we read to the informants the name of 10 plants and asked them to tell us whether they knew the plant, and–if so- whether the plant had a medicinal use. We created a knowledge score corresponding to the number of plants with medicinal use reported by the informant. The second test measured individual hunting knowledge and consisted in the identification of game animals: we presented informants with stimuli from known origin (i.e., the call of a bird, a skull of a monkey) and asked them to identify the species presented. We evaluated informants by contrasting their responses with information from known origin.

The following tests measured individual skills or practices that, according to our ethnographic information, embody local knowledge. Thus, to measure individual skills in the use of medicinal plants, we asked informants to report the last time they had prepared the plant remedies they listed in the medicinal plants identification test. We created a score on skills using medicinal plants that accounts for the total number of medicinal uses reported by the informant (from the 10 selected plants) and the last time they were used. To assess hunting skills, we asked informants to self-report on hunting frequency, weapons used, and success with difficult-to-catch prey (i.e., sun bear for Punan, tapir for Tsimane’, and wild boar for Baka). The hunting skills score was created by assigning points for each self-reported skill. The protocol for the tests used for each country can be found in http://icta.uab.cat/Etnoecologia/lek.

### Verbal working memory

We drew on classic tests based in word recall [52] to develop three culturally-specific but cross-culturally comparable tasks to assess verbal working memory. The task consisted of a multi-trial free recall of words appropriated to each cultural setting. Such tasks are considered culturally fair methods for assessing cognitive functioning in minority populations [53].

To select culturally and linguistically meaningful words for individuals, we first elicited lists of familiar items in each cultural context. We did so by conducting free-listings [54]. Specifically, in each society we asked about 20 respondents to list all the items they could think of in each of four semantic categories: a) medicinal plants, b) game, c) wild edibles, and d) locally valued market-items. We calculated the saliency of items listed, i.e., an index that takes into consideration the frequency and order in which items were listed [55, 56]. To construct culturally appropriated but cross-culturally comparable tasks, for each society we chose the exact word used to designate the four most salient items from each semantic category (4 categories * 4 items = 16 words).

Data were collected in individual sessions, consisting of three trials with the same informant. Local assistants read a list of words to participants in their local language. The list of 16 common words was randomized and all informants were presented with the same ordered-list in all the trials. Participants were then asked to recall and repeat back the words in the list without any particular mention to order.

In Trial 1, the assistant explained: *"I am going to read you a list of common words*. *I would like you to remember as many as possible*.*"* Then he read the list of words at a rate of one word every two seconds. After reading was finished, the assistant asked *"Tell me as many of the words on the list as you can remember*.*"* As informants listed words, we noted them down keeping the enumeration order (i.e., 1 for the first item the person remembered, 2 for the second, and so on). If the informant said a word that was not on the list, we marked a cell named "Other word"; this cell was marked as many times as other words were said. If the informant repeated a word, we marked the enumeration order as many times as the word was repeated.

When the informant could not recall any more words, we started Trial 2, in which the entire list was read again in the same order and speed as previously. Again, we asked informants to recall the items on the list and noted the answers. After Trial 2, a 20 minutes interval was held, a time period during which researchers stayed with the interviewee doing other tasks before proceeding to Trial 3. For Trial 3, the assistant said to the informant: *"Now we are going to go back to the list I read to you earlier*. *I need you to try and remember the words on this list and tell me what they were*.*"* Again researchers noted all the words recalled by the informant in the recall order.

Performance on the memory test was measured with a number of outcomes regarding accurate recall, inaccurate recall, and organization recall [52]. Accurate recall refers to the person’s ability to correctly recall words in the list, and in our case included the total number of correct words recalled across the three trials and in trials 2 and 3, as well as the number of words learned across trials (See Table 1 for specific variables and their definitions). Higher scores in accurate recall variables reflect better performance. Inaccurate recall refers to deviations from the original list, including repetitions of correct words, ‘recall’ of non-listed words, or inconsistency in recalling words between trials. Higher scores in inaccurate recall variables reflect worse performance (i.e., more recall errors). Last, organizational recall refers to variables that have been consistently found to help organize the cognitive process. Such variables include primacy and recency (or the ability to recall words listed first and last), semantic clustering (or the number of consecutively recalled words from the same semantic category), serial clustering (or the number of words recalled in the same order as presented), and primary memory (or the ability to recall the last word and 1-back positions during Trial 1) [29].

**Table 1:** Description of the explanatory variables used, by schooling.

Variables	Categories	Total	Schooling	No schooling
***Socio-demographic characteristics***				
Society	Baka	24	7	17
	Punan	25	18	7
	Tsimane’	45	23	22
Sex	Women	44	18	26
	Men	50	28	22
Age group	< = 30	45	29	16
	>30 & < = 45	33	12	21
	>45	16	5	11
***School-related skills***				
Numeracy	None	47	12	35
	Some	44	32	12
Literacy	None	65	24	41
	Some	28	21	7
***Local ecological knowledge and skills***				
Medicinal plants knowledge	Number of medicinal plants recognized (from a list of 10)	5.78	5.00	6.56
Hunting knowledge	Number of game stimuli recognized (from a list of 10)	5.13	5.15	5.10
Medicinal plants skills	Index of the number of medicinal uses known and the last time of use.	6.41	5.20	7.61
Hunting skills	Index capturing informant’s ability to put hunting knowledge into practice	3.77	4.04	3.51
		***94***	***47***	***47***

### Data analysis

We start the analysis by providing some descriptive statistics and assessing potential differences on measures of performance on a verbal working memory task associated to socio-demographic characteristics of the informant (i.e., studied society, sex, and age group). Such tests were conducted to assess the reliability of data. We then conducted a series of analytical tests to determine whether verbal working memory relates to a) schooling and skills typically learned in school (literacy, numeracy) and b) local ecological knowledge and skills related to medicinal plants and hunting. For variables with two levels (sex, literacy, and numeracy) we used a Wilcoxon rank-sum test. For variables with more than two levels (society, and age group), we used a one-way ANOVA. Finally, a set of correlations were run to determine the relation between an individual's local knowledge and skills and performance on the memory task. We first tested whether data were normally distributed with a Shapiro-Wilk test of normality. We used Pearson product-moment correlations for variables normally distributed and Spearman's rank-order correlations for variables non-normally distributed.

Our descriptive statistics show that younger individuals had higher levels of school attendance. To test whether there is an effect of age independent of schooling, we also ran a set of multivariate regressions. Specifically, we ran a Poisson multivariate regression for each of the variables derived from the verbal-memory task as dependent variable. An OLS model was used for the variable *Learn slope*. As explanatory variables we included 1) society’s dummies, 2) a binary variable that captures whether the person is a man or a woman, 3) a set of dummies for the age categories, and 4) a binary variable that captures whether the person had attended school or not. For statistical analysis we used Stata. Data are available in S1 Dataset.

## Results

### Description of the data

Despite external efforts to provide formal education to people in the studied societies, school levels continue to be low. Thus, only half (n = 47) of all the participants in our entire sample had attended school at some point in their life, of which 29 (62% were men) and 18 (38%) were women. Among the three societies, the lowest education levels were found among the Baka, and the highest among the Punan. Overall, younger participants have higher levels of education than older participants (Table 1). The studied sample has weak literacy and numeracy skills: only 28 informants were able to read a simple text and 44 were able to add (Table 1). However, schooling is apparently not the only way of acquiring literacy and numeracy: from the 28 who could read, seven had not attended school and from the 44 informants who could add, 12 had never attended school. Despite those inconsistencies, a series of one-way ANOVA tests showed that schooling was associated in a statistically significant way to literacy and numeracy.

Informants were able to recognize 5.78 (SD = 5.00) medicinal plants and 5.13 animals (SD = 5.15). The average score in our medicinal plant’s skill test was 6.41 and the score in hunting skills was 3.77. Informants who had attended school recognized less medicinal plants and had lower levels of medicinal plants skills (5.00 and 5.20) than informants who had not attended school (6.56 and 7.61), both differences being statistically significant (p <.001) in a t-test. Hunting knowledge and skill scores do not seem to vary depending on schooling.

On average, each informant was able to accurately recall 30.4 words across the 3 Trials (10.7 in Trial 2 and 11.5 in Trial 3), with a learning slope of 1.6 (Table 2). Typically, correct words were listed only once (i.e., not much repetitions), but on average across the three trials each informant listed 3.4 words that were not in the list. Regarding the way people organize their recall, we found that, across the 3 trials, the percentage of words recalled, from the first 4 words (23%) was higher than the percentage of the last 4 words listed (19%) and that semantic clustering occurred more often than serial clustering (0.16). 27% of the people in the sample could not recall any of the two last words listed in Trial 1, whereas 23% could recall the last two words; the remaining 50% could recall only 1 of the two lasts words.

**Table 2:** Variables derived from the verbal memory task (n = 94).

Variable	Definition	Mean	SD	Min	Max
***Accurate recall***					
Total recall	Total number of correct words recalled on the three consecutive trials (0 to 48)	30.4	7.06	12	46
Immediate recall	Total number of correct responses made in remembering the list during Trial 2 (0 to 16)	10.7	2.98	3	16
Delayed recall	Total number of correct responses made in remembering the list during Trial 3 (0 to 16)	11.5	2.88	4	16
Learn slope	Average number of words learned in each trial	1.6	1.36	-1.5	4
***Inaccurate recall***					
Repetitions	Number of times correct words were repeated in a Trial (sum across the three trials), corrected for the total number of words recalled on a trial.	0.04	0.04	0	0.17
Intruders	Number of out-of-the-list words mentioned in any of the three trials	3.4	3.6	0	13
Inconsistency	Number of times the person failed to recall a word on a later trial when it had been recalled on an earlier trial (controlling for the total number of words recalled).	0.16	0.12	0	0.55
***Organization recall***					
Primacy	Share of words recalled, across the 3 trials, from the first 4 words, considering only the total number of words recalled by the person.	0.23	0.5	0.12	0.44
Recency	Share of words recalled across the 3 trials, from the last 4 words, considering only the total number of words recalled by the person	0.19	0.6	0.6	0.41
Semantic clustering	Number of consecutively recalled words from the same semantic category, corrected by total number of correct words listed	0.29	0.11	0.05	0.53
Serial clustering	Number of words recalled in the same order as presented, corrected by total number of correct words listed	0.16	0.07	0.07	0.44
Primary memory	Share of words recalled correctly in the last word and 1-back positions during Trial 1, corrected by total number of correct words listed in Trial 1.	0.12	0.09	0	0.5

To assess the accuracy of the data, we examined potential differences in memory related variables according to socio-demographic characteristics of the sample (Table 3). Potential differences across the three societies were examined with a series of one-way ANOVA tests. We found statistically significant differences in *delayed recall* (F(2,91) = 3.19, p = .046) and *learning slope* (F(2,91) = 5.35, p = .006). A Tukey post-hoc test revealed that *delayed recall* and *learning slope* were statistically significantly higher among the Punan compared to the Tsimane’ (1.59 ± 0.70, p .07 for *delayed recall* and 0.92 ± 0.32, p .01 for *learning slope*) and the Baka (1.76 ± .80, p .08 and 1.09 ± 0.37, p .01, respectively). Among the variables that measure inaccurate recall, we found lower *repetitions* among the Tsimane’ (F(2,91) = 2,71, p = .07) and higher *inconsistency* among the Baka (F(2,91) = 2,58, p = .08). Last, the Punan also seem to exhibit larger *primacy effects* (F(2,91) = 4.48, p = .01), but lower *serial clustering* (F(2,91) = 5.96, p = .004) than the Baka and the Tsimane’. Results of a Wilcoxon rank-sum test between women’s and men’s scores suggests no statistically significant differences in our proxies for accurate and inaccurate recall. *Primacy effects* (Z = -1.895, p = 0.06) and *serial clustering* (Z = -2.141, p = 0.03), however, are higher among men. When dividing the sample according to age categories, results from a one-way ANOVA test suggest that people over 45 years of age listed more *intruder* words than people in the other two categories (F(2,91) = 11.84, p <.0001), and had lower levels of *serial clustering* (F(2,91) = 2.75, p = .07). None of the other variables analysed showed statistically significant differences between people of different age categories.

**Table 3:** Differences in variables derived from the verbal-memory task, by informant’s socio-demographic characteristics.

	Society			Sex		Age Group		
	Baka	Punan	Tsimane’	Women	Men	< = 30	>30 & < = 45	>45
***N***	24	25	45	44	50	45	33	16
***Accurate recall***								
Total recall	29.54	31.84	30.04	29.32	31.34	30.62	30.85	28.81
Immediate recall	10.17	11.12	10.67	10.14	11.12	10.56	10.91	10.44
Delayed recall	10.92	12.68**	11.09	11.36	11.56	11.64	11.64	10.62
Learn slope	1.23	2.32***	1.4	1.77	1.45	1.61	1.67	1.44
***Inaccurate recall***								
Repetitions	0.05	0.05	0.03*	0.05	0.04	0.04	0.05	0.04
Intruders	2.91	3.00	3.95	3.45	3.42	1.96	4.03	6.38***
Inconsistency	0.21*	0.14	0.15	0.17	0.16	0.17	0.14	0.18
***Organization recall***								
Primacy	0.24	0.21**	0.24	0.22	0.24*	0.24	0.23	0.21
Recency	0.21	0.19	0.19	0.19	0.20	0.21	0.19	0.18
Semantic clustering	0.27	0.31	0.29	0.29	0.30	0.29	0.28	0.30
Serial clustering	0.17	0.13**	0.18	0.15	0.18**	0.17	0.18	0.13*
Primary memory	0.14	0.14	0.10*	0.13	0.11	0.11	0.14	0.11

*, **, and *** significant at ≤0.10, ≤0.05, and ≤0.01, respectively using one-way ANOVA tests.

### Memory, school and school-related skills

We next examined whether our memory related variables varied according to school and school-related skills with a series of Wilcoxon rank-sum tests (Table 4). Our analysis provides no evidence of differences in accurate recall in relation to participant attendance to school or the acquisition of school-related skills. We found more *intruders* in the lists of informants without any schooling (Z = 2.858, p = 0.004), and without literacy (Z = 1.670, p = 0.09) or numeracy (Z = 1.673, p = 0.09); but we found no differences across the other two variables that proxy inaccurate recall. We found important differences in proxies of organization recall. People who had attended school displayed *higher primacy* (Z = -2.101, p = 0.04) and *recency* (Z = -2.453, p = 0.01) effects and higher levels of *serial clustering* (Z = -2.600, p = 0.009) but lower scores in *primary memory* (Z = 1.791, p = 0.07). Results testing the association between numeracy and the different variables calculated resemble results found when testing the variable schooling. Among the different variables tested, *primary memory* seems consistently higher among those without schooling, literacy or numeracy.

**Table 4:** Differences in variables derived from the verbal-memory task, by school and school-related skills.

	Schooling		Literacy		Numeracy	
	None	Some	None	Some	None	Some
***N***	48	46	65	28	47	44
***Accurate recall***						
Total recall	30.27	30.52	30.10	30.79	30.30	30.65
Immediate recall	10.79	10.52	10.63	10.68	10.79	10.64
Delayed recall	11.40	11.54	11.21	11.93	11.34	11.57
Learn slope	1.66	1.54	1.47	1.87	1.59	1.56
***Inaccurate recall***						
Repetitions	0.04	0.05	0.04	0.05	0.04	0.05
Intruders	4.41***	2.41	3.88*	2.50	4.09*	2.64
Inconsistency	0.16	0.17	0.16	0.16	0.16	0.16
***Organization recall***						
Primacy	0.22	0.25***	0.23	0.23	0.22	0.24**
Recency	0.18	0.21**	0.19	0.21*	0.18	0.21**
Semantic clustering	0.31*	0.27	0.27	0.27	0.30*	0.28
Serial clustering	0.14	0.19***	0.16	0.17	0.15	0.18*
Primary memory	0.13*	0.10	0.14***	0.08	0.13**	0.10

*, **, and *** significant at ≤0.10, ≤0.05, and ≤0.01, respectively using one-way ANOVA tests.

Since, overall, schooled individuals tended to be younger than unschooled ones, we ran multivariate regressions to test for the potential confounding effects of age and schooling. The results (not shown) are substantially no different than the results of bivariate analysis. For example, results of multivariate analysis confirm that people without schooling listed more *intruders* than people with schooling (p<0.01), even after controlling for their age category. The same results also show that people in higher age categories listed more *intruders* (p<0.01), even after controlling for school attendance.

### Memory, local ecological knowledge and skills

In our final test, we examined whether our memory-related variables are associated to local ecological knowledge and skills (Table 5). Our analysis provides no evidence of correlation between measures of accurate recall and measures of local ecological knowledge and skills. There was a statistically significant and positive correlation between the number of *intruder* words a person listed and three of our variables for local knowledge: medicinal plants knowledge (rho = 0.253, n = 86, p = .02), medicinal plants skills (rho = 0.354, n = 86, p<.001), and hunting skills (rho = 0.226, n = 87, p = .04). However, we found no association with *repetitions* and *inconsistency*. Regarding the organization of recall, we found that people with higher medicinal plants knowledge (r = -0.300, n = 86, p = .005) and medicinal plants skills (rho = -0.334, n = 86, p = .001) had lower *recency*, and people with higher medicinal plants knowledge also had lower *serial clustering* (rho = 0.245, n = 86, p = .02).

**Table 5:** Differences in variables derived from the verbal-memory task against local ecological knowledge and skills.

	Medicinal plants knowledge	Hunting knowledge	Medicinal plants skills^†^	Hunting skills^†^
***N***	86	87	86	87
***Accurate recall***				
Total recall	-0.031	0.002	-0.033	-0.082
Immediate recall	0.024	0.016	0.023	0.065
Delayed recall	-0.029	-0.029	-0.057	-0.014
Learn slope	0.049	-0.049	-0.050	-0.102
***Inaccurate recall***				
Repetitions^†^	0.015	-0.007	-0.072	-0.074
Intruders^†^	0.253**	0.013	0.354***	0.226**
Inconsistency^†^	-0.077	0.014	-0.081	-0.031
***Organization recall***				
Primacy	-0.071	0.041	0.020	0.094
Recency	-0.300**	0.135	-0.334**	-0.062
Semantic clustering	-0.007	-0.135	-0.121	-0.054
Serial clustering^†^	-0.245*	0.122	-0.094	0.058
Primary memory^†^	0.009	-0.058	-0.036	0.017

*, **, and *** significant at ≤0.10, ≤0.05, and ≤0.01, respectively using Pearson correlations.

^†^ Variables not normally distributed. Correlations using those variables are Spearman's rank-order correlations.

## Discussion

The goal of this work was to assess whether the acquisition of two forms of knowledge (schooling and local ecological knowledge) relate differently to cognition, for which we compared measures derived from a set of verbal memory tasks across people with different levels of schooling and school-related skills and with different levels of local ecological knowledge and skills. Despite working with three different forager societies, we found overall comparable results in our evaluation of verbal working memory across men and women and people from different age categories. The only significant difference here was that the Punan of East Kalimantan, who seemed to display lower levels of *primacy* and *serial clustering* than the Baka and the Tsimane’, although they seem to have similar levels of accurate recall. Data from this study reveal three main findings. First, people with and without schooling have similar levels of accurate and inaccurate recall, although they differ in their strategies used to organize recall. Second, from the two school-related skills tested, numeracy relates to memory in the same way as schooling does, whereas literacy showed weaker associations. Third, contrarily to schooling and school related activities, the level of local ecological knowledge or skills of an individual does not seem to be related to accurate recall, inaccurate recall, or recall organization. We centre the discussion on these three findings.

### Memory and schooling

Irrespectively of whether they had attended school or not, people in our sample have similar levels of accurate and inaccurate recall. Interestingly, however, they seem to achieve those results using different strategies: people with schooling seem to have higher *primacy*, *recency*, and *serial clustering*, but lower *primary memory* and *semantic clustering* than people without schooling. Better performance on primacy, recency, and serial clustering suggests a larger reliance on rote learning amongst those with schooling, whereas better performance on semantic clustering suggests a larger reliance on semantically meaningful categories to organize recall amongst those who had never attended school.

The relation between schooling and cognitive status is well-known, with several studies showing that schooling has lasting effects both on cognitive [57] and non-cognitive skills [3], even moderating the trajectory of cognitive change as people age [58]. Our findings, however, challenge such previous results. The most plausible explanation for such contrasting finding lies in the type of populations being studied.

Most previous studies analysing the effects of schooling or education on cognition have to date taken place in Western societies [19, 20, 30], where schooling and the skills learned at school are considered fundamentally important for the future work, economic, and social possibilities (and hence well-being) of the individual. In such settings, illiteracy or lack of schooling is often cofounded with many other social factors (i.e., poverty and discrimination) [59]. In contrast, we set our study among hunter-gatherer societies in which subsistence and well-being do not depend on schooling but rather on local ecological knowledge. Such dissimilarities in the type of population being studied might explain the contrasting findings in two non-antagonist ways. On the one side, as psychologists suggest, it might just be that people of different cultural backgrounds process information differently [53, 60], implying that basic cognitive abilities like attention, executive control, or short-term memory can be affected by individuals’ experience in the world (i.e., by culture) [61]. As the three studied societies resemble each other more than they resemble Western societies, they might experience similar effects of schooling on cognition. On the other side, the explanation might also lie in the use of specific cognitive skills common during preliteracy. Anthropological studies have shown that preliterate societies, like those studied here, possess complex classification systems that guide cognition [62–64]. Furthermore, some recent studies also suggest differences on the type of learning strategy used in such societies in the absence of schooling [23]. As our memory task included words that could be organized in local emic classifications, it is possible that -in such circumstances- people could use retrieval strategies more familiar to them. Such an explanation is in line with Cole and Scribner’s [26] finding that when non-schooled children were tested using a list of objects presented in a meaningful way, they recalled them easily by drawing the connections through the story.

### Memory and school related skills

Our second main finding relates to the different effects of literacy, numeracy, and schooling on memory. Researchers have argued that schooling and the skills learned in school might affect cognition differently, as the process of learning to read might train specific abilities, such as explicit phonological awareness, spatial perception, fine motor skills, and the attainment of grapheme to phoneme correspondence, which might potentially affect brain functioning [19]. Our findings support the hypothesis that the effects of schooling and school-related skills might be different, but in an unexpected direction. We found that numeracy relates to memory in the same way that schooling does, whereas literacy showed weaker associations.

Previous research has found that illiterate people generally perform less well than literate people on conventional neuropsychological memory tests including wordlist learning and recall (the task used here), but also on story learning and recall, verbal paired associates, digits backwards, letter-number sequencing tasks, and complex figure drawing (see [4] and references within). The performance of illiterates seems to approach that of literates only in object memory and wordlist recognition memory [4]. According to Ardila and colleagues [19], such differences in recall between literate and illiterate people can be explained because illiterate individuals have inefficient encoding and retrieval strategies or poor organization of the material to be memorized, which makes it difficult to support a relatively active and effortful cognitive process such as free recall. Our data do not support such findings, as there were no differences between literate and illiterate participants except for in primary memory.

The difference can also be due to low levels of literacy (even many literate informants have difficulty reading a short sentence). However, an important implication of our finding is that, in the studied populations, differences in verbal working memory performance should be attributed to school attendance per se, and not to literacy.

### Memory and local ecological knowledge and skills

The third important finding of this work relates to the lack of association between our measures of local ecological knowledge and skills and our measures of accurate recall, inaccurate recall, and recall organization on the other. Why, in contrast to schooling, is local ecological knowledge not associated to verbal working memory? Researchers studying the process through which local ecological knowledge is acquired argue that the acquisition of such types of knowledge is largely based on emulation, context and meaning [65, 66], rather than on verbal communication and memorization. The acquisition of local ecological knowledge generally involves learning mechanisms that are more procedural, pragmatic and sensory oriented [67, 68]. For example, Schank and Abelson [69] argue that stories about one's own experiences, and the experiences of others, are the fundamental constituents of human memory, knowledge, and social communication, implying that decontextualized information is hard to remember. Worldwide, the introduction of western-style schooling in non-western societies has impacted on the value given to local ecological knowledge, due to the duality between scientific and traditional knowledge, with the former highly predominating in formal schooling. In recent work, Berl and Hewlett [23] notice that contrarily to Ngandu schooled children, Aka adults and western children, non-schooled Aka children rely more on emulation than on over-imitation as a learning strategy. In other words, the acquisition of local ecological knowledge and skills is often embedded in everyday activities, and does not necessarily depend on oral communication [23]. Because the acquisition of local ecological knowledge might be more independent from verbal communication than school-related learning, it is not surprising that we do not find a relation between levels of local ecological knowledge and measures of verbal memory.

## Conclusion

Our finding that people from forager societies with and without schooling have similar levels of accurate and inaccurate recall supports the idea that although school attendance might relate to the performance of some cognitive tests, this might not necessarily manifest in daily performance (see also [70]), mostly because people might use different strategies for subsistence and livelihood. From this finding, we conclude that while schooling seems to favour some organization strategies this might come at the expense of some other organization strategies. In other words, the trade-off of schooling might go beyond the fact that time and resources spent in school detract from time and resources spent learning other forms of knowledge [14–16]; such trade-off might also affect cognition.

## Supporting Information

S1 Dataset Raw data used in the analysis (n = 94). (XLSX)
